# Genomic Structure of the Porcine *CYP19* Locus and Expression of the *CYP19A3* Paralog

**DOI:** 10.3390/genes12040533

**Published:** 2021-04-06

**Authors:** Jens Vanselow, Alan J. Conley, Cynthia J. Corbin, Trish Berger

**Affiliations:** 1 Leibniz Institute for Farm Animal Biology (FBN), Wilhelm-Stahl-Allee 2, 18196 Dummerstorf, Germany; 2 Department of Population Health & Reproduction, School of Veterinary Medicine, University of California, Davis, CA 95616, USA; cjcorbin@ucdavis.edu (A.J.C.); ajconley@ucdavis.edu (C.J.C.); 3 Department of Animal Science, College of Agricultural and Environmental Sciences, University of California, Davis, CA 95616, USA

**Keywords:** transcription, RT-qPCR, 5´RACE, hypothalamus, testis, ovary

## Abstract

Proper, tissue-specific regulation of *CYP19*, the gene encoding aromatase, the key enzyme of estrogen synthesis, is essential for reproductive processes. Here, we analyzed transcriptional regulation of the porcine *CYP19* in female and male gonads and brain by 5’RACE and RT-PCR and comprehensively mapped the pig *CYP19* locus by in silico analysis. Our data revealed that the complete locus, including three paralogous copies, *CYP19A1*, *CYP19A2* and *CYP19A3*, spans approximately 330 kb of the porcine chromosome 1. The locus also harbors the first exon of the Gliomedin gene (*GLDN*) in reverse orientation. Only transcripts of the *CYP19A3* paralog were substantially expressed in gonads and hypothalamus. We identified *CYP19A3*-associated untranslated exons approximately 160 kb and 50 kb distal from the first codon. The 5´ untranslated regions of transcripts were derived from either a proximal or from one of these distal untranslated exons. Transcripts including only untranslated exons could be amplified from testis, thus suggesting long non-coding transcripts. The data revealed an additional layer of complexity in the regulation of the porcine *CYP19* locus. Tissue-specific expression is not only achieved by tissue- and stage-specific expression of the three different *CYP19* paralogs, but also by directing the expression of *CYP19A3* from different, proximal and distal promoter regions.

## 1. Background

Expression of *CYP19*, the gene encoding aromatase, the key enzyme of estrogen synthesis, is essential in regulating female as well as male reproductive processes. Transcripts, proteins and enzyme activity are present in many different male and female tissues, including the ovaries, placenta and testis [1,2,3]. Other sites of *CYP19* expression include the adrenal gland [1,4], adipose tissue [5,6,7] and brain [3,4,8,9,10]. In particular, in certain regions of the male brain, locally produced estradiol is believed to play an important role in the development of sexual behavior based on the results of studies in mice and sheep [11,12]. Additionally, aberrant or ectopic expression of human *CYP19* is thought to induce pathogenic processes such as tumorigenesis [13,14,15]. Therefore, it is not surprising that tissue-specific transcription of this gene is tightly regulated. This is achieved by the utilization of different, sometimes very distally (relative to the first codon) located, promoter regions, initially recognized in studies that sequenced *CYP19* transcripts isolated from different human tissues. The use of alternative, tissue-specific promoters results in transcripts with an identical coding sequence comprising nine different exons (exons II to X in human), but different 5´ untranslated regions/exons (5´UTR) [7,16]. Similar mechanisms of transcriptional regulation of *CYP19* expression have been reported in other mammalian species, particularly in the bovine [4,17]. Interestingly, these promoter regions and the corresponding 5´UTRs found in tissue-specific transcripts appear to be conserved among species in some cases but not others. The most proximal promoter, utilized for *CYP19* expression in ovarian granulosa cells during late folliculogenesis, is similar in most mammalian species studied to date. In contrast, the more distally located, placenta-specific human and bovine promoters are in non-homologous genomic regions [4,17], as is the ovine placental promoter [18], despite the close evolutionary relationship between cattle and sheep. In addition to this complexity of regulatory sequences, the existence of a second genomic copy of *CYP19* was reported in the bovine [19]. Corresponding transcripts were detected in placental tissue, however, with relatively low abundance. Together with the finding that these transcripts were truncated and included substantial deletions and mutations in the reading frame, this copy was interpreted to be a non-functional pseudogene, *CYP19ψ*. However, in light of our current knowledge of the important roles of long and short non-coding RNAs, a regulatory role of these truncated *CYP19ψ* transcripts remains conceivable [20]. The sheep genome contains a second partial or complete copy of *CYP19*, although only one copy is transcribed and encodes a functional protein [21].

Given the evidence of at least partial duplication of *CYP19* in livestock, it is noteworthy that the bovine genome also includes three paralogous copies of another important steroidogenic gene, the *CYP17A1* gene encoding P450c17, the key enzyme of androgen synthesis. However, only one of these copies was found to be actively transcribed in steroidogenic tissues. Both of the other copies of *CYP17A1* appeared to be silenced by hypermethylation of their promoter regions [22]. This suggests that gene duplication has occurred quite frequently during evolution in these species and that activation or silencing of specific paralogs might be regulated by long-term epigenetic mechanisms such as DNA methylation.

In the pig, however, early studies clearly indicated that different functional aromatase isoforms are present in the ovary and placenta [23]. In fact, these tissue-specifically expressed isoforms could be assigned to paralogous copies of *CYP19* in the porcine genome, named types I to III [24,25]. After the discovery of two copies of *CYP19* in Pecari species, it became evident that one or more rounds of *CYP19* gene duplication must have occurred during the evolution of suiform species, with the unique feature compared with other mammalian species that these copies are expressed and encode multiple functionally distinct aromatase enzymes [26,27].

Two different *CYP19* genes, *CYP19a* and *CYP19b*, with a distinct tissue-specific regulation of expression, were also found in fish [28,29,30].

The role and regulation of different *CYP19* gene copies has been studied most extensively in the pig. Expression of one of these copies was found in the male and female gonads, adrenal gland and hypothalamus [8,31]. This copy is identical to the type I gene now known as *CYP19A3* (NCBI database). Transcripts of a second copy were identified in placental tissue, which is identical to the type II gene now referred to as *CYP19A2*. Transcripts of the third copy, type III or *CYP19A1*, are expressed by porcine blastocysts during implantation, consistent with the concept that estradiol is an important signal during the early embryo–maternal dialog [32]. In a recent paper, the *CYP19A2* copy (the placental paralog based upon the deleted sequence, as shown in Figure S1 in [33]) was knocked out using CRISPR/Cas9 technology and somatic cell nuclear transfer, with the result that the maintenance of pregnancy beyond the 30^th^ day was completely disrupted [33]. The isoforms of aromatase encoded by the *CYP19* gene paralogs show considerable functional differences in catalytic activity and sensitivity to imidazole inhibitors resulting from significant divergence of their amino acid sequences. The coding sequences (CDS) of *CYP19A1* and *CYP19A2* mRNAs include 1509 nucleotides (nts), thus encoding proteins of 503 amino acids in length. This is identical to *CYP19A1* in other mammalian species. In contrast, the CDS of *CYP19A3* transcripts spans only 1503 nts, thus encoding a protein of only 501 amino acids in length. As first reported by Corbin et al. [23], porcine placental aromatase was almost 10-fold more active in estrone synthesis than aromatase in granulosa cells. Hypothetically, the highly active placental isoform may help to protect the maternal tissues and female siblings from male fetal androgens by their conversion into estrogens. Tissue-specific occurrence of the different porcine *CYP19* copies is summarized in Table 1.

In an early study [31], the authors presented some evidence that transcription of *CYP19* mRNA in the ovary and testis, although including an identical CDS (*CYP19A3*), might be initiated from different promoter regions. In combination with the tissue-specific expression of the three *CYP19* paralogs in porcine species, this would result in an even more complex transcriptional regulation of the *CYP19* gene than in other mammalian species that have only one functional genomic copy.

The present study was conducted to further elucidate the organization and complex regulation of the porcine *CYP19* locus comprising three functional paralogs with a highly complex stage- and tissue-specific expression pattern, focusing on the expression of the *CYP19A3* copy in the female and male gonads and hypothalamus. Tissue-specific transcripts were isolated with 5´RACE and RT-PCR and the corresponding genomic sequences were mapped by in-silico analysis to reconstruct the complete porcine *CYP19* locus.

## 2. Materials and Methods:

### 2.1. Animals and Tissue Collection

Hypothalami, ovaries and testes (after removal of tunica albuginea and mediastinum tissue) were obtained from 5–6-month-old (peripubertal age) females and males. Tissues were flash frozen in liquid nitrogen immediately after isolation. In addition, hypothalami and testes were isolated from 6-week-old boars similarly flash frozen on dry ice. All samples were obtained from commercial crossbred pigs and tissues were stored at −80 °C prior to RNA isolation.

### 2.2. RNA Preparation, 5´ RACE, cDNA Synthesis and RT-PCR

RNA isolation was performed using the innuPREP RNA Mini Kit (Analytik Jena, Germany) or Trizol (Thermo-Fisher, Waltham, MA, USA) according to the manufacturers´ protocol and quantified with a NanoDrop1000/2000 Spectrophotometer (Thermo Scientific, Bonn, Germany).

The rapid amplification of cDNA ends (RACE) procedure was performed using a 5′/3′ RACE Kit, 2nd Generation (Roche Diagnostics GmbH, Mannheim, Germany, cat #03 353 621 001) following the manufacturers’ recommended procedures. PCR products were purified using a High Pure PCR Product Purification Kit (Roche Diagnostics GmbH, cat #11 732 668 001) and sequenced. First-strand synthesis and amplification used nested gene-specific primers (Table 2), the design of which was based on previously published sequences [1,8,31].

Complementary DNA for RT-PCR analysis was synthesized with a mix of oligo-dT and random primers using the SensiFAST cDNASynthesis Kit (Bioline, Luckenwalde, Germany) or the Revertaid First Strand cDNA Synthesis Kit from 200 ng RNA.

Fragments of *CYP19* and *GLDN* transcripts were amplified with specific forward and reverse primers (Table 3) with Taq DNA Polymerase (Cat# EPTQA025, MP Biomedicals) or AmpliTaq Gold DNA Polymerase (Cat # 4309155 for cDNA MasterMix, Applied Biosystems).

PCR products were analyzed on 2–3% agarose gels containing 0.02% SYBR Safe gel stain or 0.02% Roti-GelStain (Carl Roth GmbH, Karlsruhe, Germany) with a 1 kb DNA ladder (Quick-Load Purple 1 kb Plus DNA Ladder, New England BioLabs) as size marker.

### 2.3. Sequencing of PCR Products

After visualization of the PCR products on agarose gels, representative bands were cut out and the DNA purified using QIAquick gel extraction kit (#28704, Qiagen, Germantown MD, USA). Concentration was determined using the Nanodrop and 10 µL of the DNA template at >1 ng/µL and 5 µL of 5 µM primer (forward and reverse in separate sequencing) was submitted to Genewicz (South San Francisco, CA, USA) for sequencing. Alternatively, representative PCR products were purified using the High Pure PCR Product Purification Kit (Roche Diagnostics GmbH) and directly sequenced by Microsynth Seqlab GmbH (Göttingen, Germany) by using forward and reverse primers.

## 3. Results

### 3.1. Analysis of 5´RACE Products

**Figure 1:**
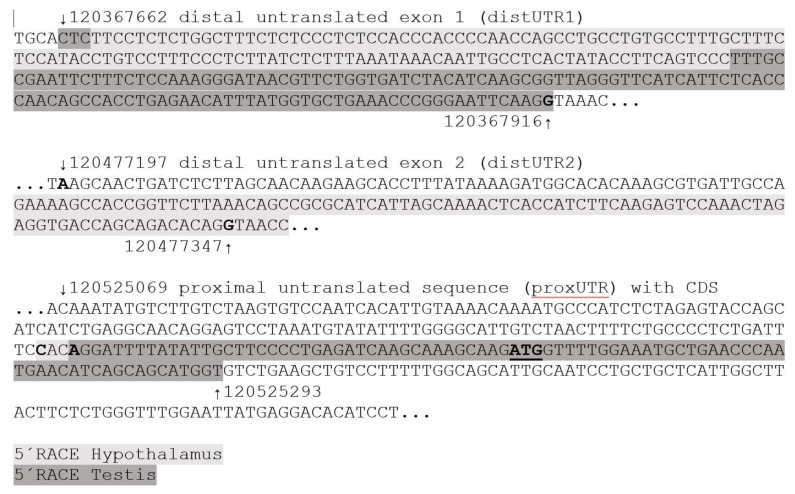
Distal and proximal untranslated regions and partial coding sequence of *CYP19A3*. Here, 5´RACE and RT-PCR products from porcine female and male gonads and hypothalami with high similarities (≥97%) were mapped to the pig genomic sequence. ↓ and ↑ indicate position numbers of selected nts in the pig genomic contig NC_010443.5 of Chromosome 1. Regions with sequence similarities to 5´RACE clones from hypothalamus (grey) and testis (dark grey) are highlighted. Presumptive splice sites and the first codon (ATG) of *CYP19A3* are printed in bold.

First, the 5´RACE products generated from the testis and male hypothalamus samples from 6-week-old boars were sequenced, revealing that the CDS was clearly derived from *CYP19A3*, with a similarity of 100% and 97% in the case of the testis- and hypothalamus-derived sequences, respectively. However, the 5´UTRs were different: 255 nts of the testis-derived sequence mapped with 100% sequence similarity between positions 120367662 and 12036716 of the porcine genomic contig NC_010443.5 from Chromosome 1 (Figure 1).

This sequence will be referred to as “distal untranslated exon 1” or as “distalUTR1” hereafter. The hypothalamus-derived sequence partially mapped to the same genomic region (120367665 to 120367793) but with less sequence similarity (95% over 128 nt), mainly due to poor sequencing quality. High sequence similarity (100% over 90 nt) was evident when the hypothalamus-derived sequence mapped to a genomic region located approximately 50 kb upstream of the first codon of *CYP19A3* (120477263 to 120477352). This sequence will be referred to as “distal untranslated exon 2” or as “distalUTR2”. Interestingly, a similar sequence was also reported as the 5´UTR of brain-derived *CYP19* transcripts from several other mammalian species, including human (D29757.1, 96% similarity over 165 nts), bovine (Z82979.1, 94% over 96 nts) and mouse (D67045.1, 92% over 166 nts).

### 3.2. Reconstruction of the Pig CYP19 Locus

**Figure 2:**
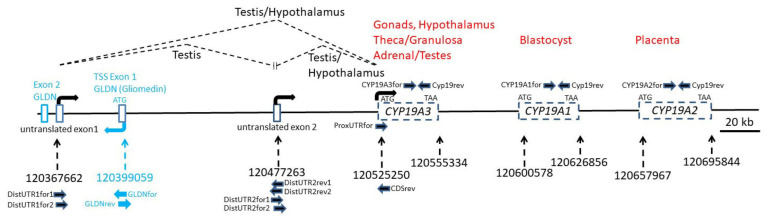
The pig *CYP19* locus. The pig genomic region harboring the *CYP19* locus and the first exon of Gliomedin (*GLDN*) was constructed by mapping published reference mRNA sequences of *CYP19A1* (NM_214429), *CYP19A2* (NM_214430) and *CYP19A3* (NM_214431), sequences from hypothalamus and testis 5´RACE clones and from RT-PCR products to the pig genomic contig NC_010443.5. Positions in NC_010443.5 are indicated with dashed arrows. Transcription start sites as indicated by RT-PCR experiments and positions of corresponding forward and reverse primers are indicated with arrows. Published sites of expression of the three *CYP19* paralogs are shown in red (see also Table 1). Splicing of untranslated exons as found by RT-PCR experiments in testis and hypothalamus is indicated with dashed lines. Positions of untranslated exons of *CYP19A3* and of coding exons within *CYP19A1*, *CYP19A2* and *CYP19A3* are indicated.

Starting with the sequence fragments of the 5´RACE products and the published sequences of the porcine *CYP19A1, CYP19A2* and *CYP19A3* reference mRNAs, we reconstructed the complete porcine *CYP19* locus with the Nucleotide BLAST tool of the NCBI database. All sequences matched to the porcine genomic contig NC_010443.5 from Chromosome 1 within a region of 328 kb length from position 120367662 to 120695844 (Figure 2).

Surprisingly, this locus also harbors the first exon including the ATG translation start site of the Gliomedin gene (*GLDN*) in reverse orientation. Contig NC_010443.5 was found to contain a very distant untranslated exon (distal untranslated exon 1) that was in both 5´RACE clones. The most distal exon is located nearly 160 kb upstream from the CDS of CYP19A3, and the second untranslated exon (distal untranslated exon 2) isolated from the hypothalamus sequence is approximately 50 kb upstream from the CDS of *CYP19A3*. Accordingly, the regulatory region of the *CYP19A3* gene comprises both untranslated exons and presumably associated promoter regions, thus extending over a considerably longer genomic area than both other *CYP19* copies (*CYP19A1* and *CYP19A2*), including their presumable 5´ regulatory regions.

### 3.3. Isolation of Tissue-Specific CYP19 Transcripts by RT-PCR

**Figure 3:**
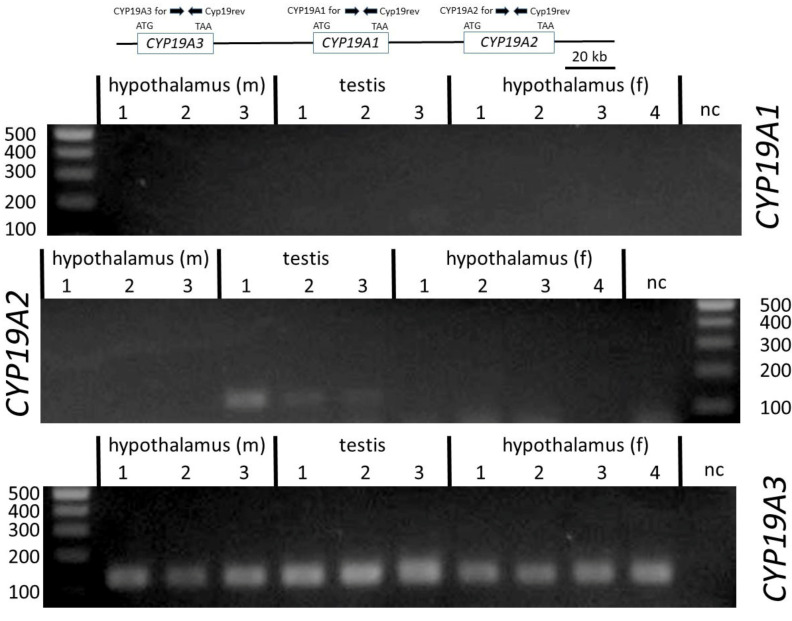
PCR products amplified with specific primers annealing to coding exons of *CYP19A1*, *CYP19A2* and *CYP19A3*. Only transcripts from *CYP19A3* were found in testis and in female and male hypothalamus from three to four individuals. *CYP19A1* transcripts were not found in any of the samples, and only a weak band of *CYP19A2* was amplified in testis of one boar (#1). Annealing positions of the respective primers are indicated in the inserted figure. f, m = female, male. Sizes of respective markers in base pairs are indicated.

To verify the sequences derived from hypothalamus and testis 5´RACE clones and to study the occurrence of *CYP19* transcript variants, forward and reverse primers from the different untranslated regions and from the CDS were designed based on sequences from the genomic contig NC_010443.5 (Table 2 and Figure 2). We also analyzed the incidence of transcripts derived from the three paralogous copies *CYP19A1, CYP19A2* and *CYP19A3*. For this, primers were derived from the respective CDS of the different published reference mRNAs NM_214429 (*CYP19A1*), NM_214430 (*CYP19A2*) and NM_214431 (*CYP19A2*). The reverse primer was selected from a region with 100% sequence similarity between all three copies. Forward primers were designed identical over 23 nts to enable similar PCR efficiencies as far as possible—however, with two nts being copy-specific at the respective 3´ ends. Length of amplicons was 136 nts. As indicated in Figure 3, only *CYP19A3* transcripts could be amplified from all testis and hypothalamic tissue samples. *CYP19A1* transcripts were not generated in any of the samples, whereas from *CYP19A2* transcripts, only in the testis of boar #1 could a weak band be generated.

**Figure 4:**
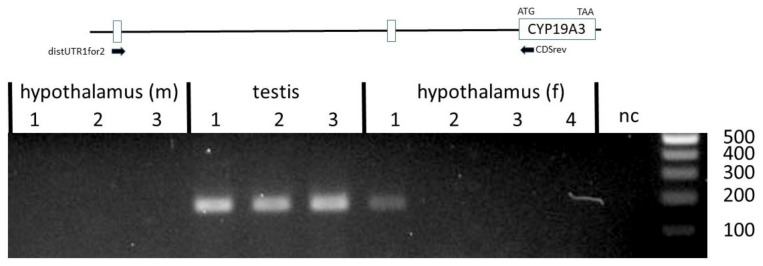
PCR products amplified with primers annealing to the distal untranslated exon 1 and CDS. A clearly visible band was found in all testis samples, a weak band only in hypothalamus of female #1. Annealing positions of corresponding forward and reverse primers in the distal untranslated exon 1 and the CDS of *CYP19A3* are shown in the inserted Figure 1. this transcript variant was detected but represented by a considerably weaker band. These transcripts were also present in both peripubertal ovarian samples based upon sequencing of isolated PCR products (not shown). Direct sequencing analysis of PCR products and the presence of only one band in the gel clearly indicated that a sequence derived from distal untranslated exon 2 was not spliced into any of these products.

Another set of primers was designed to verify if the *CYP19A3* transcripts from the testis, ovary and hypothalamus included a 5´UTR derived from the distal untranslated exon 1. Corresponding transcript variants could be clearly amplified from all testis samples (Figure 4).

Transcripts including the proximal 5´UTR derived from a genomic sequence adjacent to the CDS could be amplified from ovary, testis and male and female hypothalamus with primer ProxUTRfor. The identity of these was verified by direct sequencing of PCR products (not shown).

**Figure 5:**
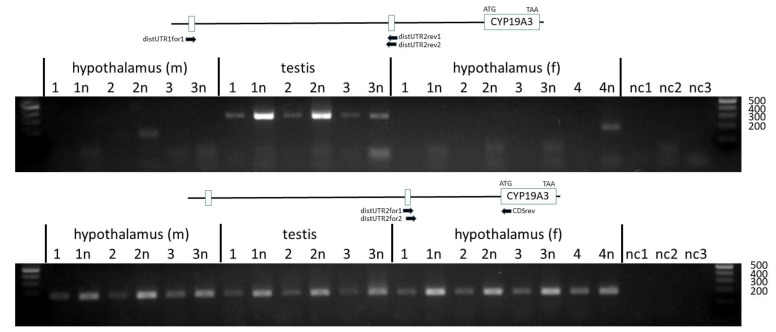
PCR products amplified with primers annealing to the distal untranslated exons 1 and 2 and to the CDS. Products Figure 2. and the CDS were found in all tissue samples. Sizes of respective markers in base pairs are indicated.

To analyze the inclusion of the distal untranslated exon 2 in *CYP19A3* transcripts, forward and reverse primers in nested positions were designed based on the sequence derived from contig NC_010443.5. These were combined with either a forward primer annealing to distal untranslated exon 1 or with reverse primer CDSrev annealing to the CDS of *CYP19A3*. As evident from Figure 5 (upper panel), transcripts including both untranslated exons were only derived from the testis but not from the hypothalamus transcripts. In contrast, products including the distal untranslated exon 2 and the CDS could be amplified from transcripts isolated from all tissue samples (Figure 5, lower panel). The identity of PCR products was confirmed by direct sequencing.

We also tested if transcripts might exist that include sequences from one or both distal untranslated exons and from the nearby first exon of the gliomedin gene (*GLDN*). However, none of these primer combinations with *GLDN*-specific primers (see Figure 2) yielded any correct products (not shown).

## 4. Discussion

Previous studies have already demonstrated the existence of three functional paralogous copies of *CYP19* [24,25]. Data from the present study, however, provide for the first time a comprehensive image of the complex porcine *CYP19* locus derived from our present knowledge of the porcine genome. The relative positions, orientations and distances of the three *CYP19* paralogs to each other are presented, although some refinement may be necessary depending on the progress and validation of the porcine genome sequencing. In this map, it is clear that, as in many other mammalian species, most of the locus comprises regulatory, non-coding sequences such as untranslated exons and associated promoters and start sites of transcription. Interestingly, more than half of the locus is covered by the *CYP19A3* copy, with its quite distal 5´ untranslated exons. Together, *CYP19A1* and *CYP19A2* occupy much less of the sequence associated with this locus. This spatial arrangement suggests a less complex regulation of these copies, which does not involve very distal multiple start sites of transcription, as found in *CYP19A3*. One might speculate that *CYP19A3* shows a more complex expression pattern in the female and male gonads, hypothalamus and adrenal gland, whereas *CYP19A1* has so far only been detected in early embryos and *CYP19A2* in the placenta [24,25]. However, further investigation is needed to determine if one or both of these *CYP19* genes might be regulated by sequences upstream of *CYP19A3*. In any case, the present map may help further studies to identify and characterize regulatory sequences of this locus, including epigenetic modification, such as DNA methylation, histone modification or chromatin packaging.

The structural overlap between *CYP19*A3 and the *GLDN* gene was an unexpected finding. The first exon of *GLDN* was identified downstream from the distal untranslated exon 1. *GLDN* encodes the gliomedin protein that plays a role in Schwann cell–axon interactions [34]. The function of this gene and its expression pattern are very different from those of *CYP19A3*, thus likely excluding any interactions between these genes. According to the NCBI database, *GLDN* is mainly expressed in the brain, colon, fat, lung and placenta, but not in the female or male gonads. In addition, we did not find hybrid transcripts of *GLDN* and *CYP19A3* exons. However, one cannot eliminate the possibility that transcription of both loci might interfere or interact in some way with each other.

Expression analysis by RT-PCR with copy-specific primers clearly confirmed data of earlier studies that transcripts of *CYP19A3* but not of either paralog, *CYP19A1* or *CYP19A2*, are generally present in female and male gonads. The presence of *CYP19A2* transcripts, which are usually expressed in the placenta [24], was observed in one of the testis samples, albeit at a very low level relative to those of *CYP19A3* (see Figure 3). Thus, our data clearly confirm differential tissue-specific expression of the three *CYP19* paralogs in male and female pigs.

In an early study, different 5´UTRs were demonstrated in transcripts from theca and granulosa cells, as compared with testes, adrenal glands and placenta, thus suggesting tissue-specific alternative splicing [31]. Data from the present study confirmed these observations, but in addition indicated that three different 5´UTRs derived from distal untranslated exons 1 and 2, and from a proximal untranslated sequence, are associated with the CDS of the *CYP19A3* paralog in the testis, ovary and hypothalamus. However, these variants appear to be differentially expressed in the testis and hypothalamus. The UTR derived from the distal untranslated exon 1 was mainly found in testis transcripts and only expressed at very low (and sometimes undetectable) levels in hypothalami, whereas the UTR from the distal untranslated exon 2 was utilized in testis as well as in female and male hypothalamus samples (see Figure 4). Interestingly, transcripts including an untranslated sequence with considerable similarity to distalUTR2 were also reported in brain-derived *CYP19* transcripts from several other mammalian species [3,4,10,35,36,37]. However, our data do not support the conclusion that this untranslated exon is preferentially or even exclusively expressed in the brain, especially as we identified corresponding transcripts with seemingly similar levels in testis as well as female and male hypothalamus samples. In any case, our data are consistent with the previous observation that *CYP19* expression is regulated differently in the hypothalamus compared with the testis [8].

Unexpectedly, however, we could not amplify transcripts including both distal 5´UTRs in addition to the first coding exon of *CYP19A3*. This combination was found in the 5´RACE sequence from hypothalamus. Neither the obligatory size analysis by agarose gel electrophoreses nor direct sequencing of selected PCR products revealed any such transcript variants. This suggests that either those variants are extremely rare or the 5´RACE clone was a partial PCR artefact. It is very unlikely that transcripts including both 5´UTRs and the CDS were not amplified due to their size, because the maximally expected size of approximately 500 nts would be clearly within the size range of the PCR system used. On the other hand, transcripts including both distal UTRs could be amplified from testis samples if forward and reverse primers were directly annealing to these sequences (see Figure 5). This may suggest that transcripts comprising both untranslated exons but without CDS are generated and may act as regulatory non-coding RNAs. In any case, our data suggest that the genomic region between positions 120367662 and 120525250 of genomic contig NC_010443.5 (see Figure 2) may harbor various regulatory sequences. It includes various start sites of transcription from two different genes, *GLDN* and *CYP19A3*, and may be the template for non-coding regulatory RNA. A more in-depth analysis of this regulatory region may bring interesting modes of transcriptional regulation of this region to light.

## 5. Conclusions

The porcine *CYP19* locus includes three paralogous copies, *CYP19A1*, *CYP19A2* and *CYP19A3*, spanning approximately 330 kb of chromosome 1. The locus also harbors the first exon of the Gliomedin gene (GLDN) in reverse orientation. Transcripts of *CYP19A3* were expressed in male and female gonads and brain primarily from two distal, as well as a proximal, promoter regions. Thus, tissue-specific transcription of the porcine *CYP19* locus is achieved by tissue- and stage-specific expression of the three *CYP19* paralogs and in addition by directing the expression of *CYP19A3* from different promoter regions.

## 6. Ethics Approval

Samples from 6-month-old female and male animals were obtained from pig carcasses during regular pig slaughtering. The animals were slaughtered in an approved abattoir (MV21212) by qualified personnel in accordance with the German animal welfare regulations (TSchlV) and the guidelines for the German initiative of animal welfare (Initiative Tierwohl). Experiments with juvenile boars was conducted in accordance with the Guide for the Care and Use of Agricultural Animals in Research and Teaching and approved by the UC Davis Institutional Animal Care and Use Committee.

## Figures and Tables

**Table 1:** Porcine *CYP19* copies and sites of expression.

NCBI Database (mRNA)	Isoform	Site of Expression
*CYP19A1* (NM_214429)	Type III [24,25]	Blastocyst [24], Embryo [25]
*CYP19A2* (NM_214430)	Type II [24,25]	Placenta [24,25]
*CYP19A3* (NM_214431)	Type I [24,25]	Ovary [24,25], Theca/Granulosa [31], Adrenal gl./Testes [31], Hypothalamus [8]

**Table 2:** Gene-specific reverse primers used for 5´RACE.

Name	Sequence	Length (nt)	Pos in NM_214431	
PCR-0	AGTTGCAGGCACTGCCAATCC	21	200	220
PCR-1	AATAGCCAGGACCTGGTATTG	21	131	151
PCR-2	GGACAGCTTCAGACACCATGCTG	23	36	58

**Table 3:** Primers used for RT-PCR.

Name	Sequence	Length (nt)	Pos in NC_010443.5		Orient.
DistUTR1for1	TGGCTTTCTCTCCCTCTCCA	20	120367673	120367692	for
DistUTR1for2	ACATCAAGCGGTTAGGGTTCA	21	120367836	120367856	for
GLDNfor	AGCACATCCGCACAGAGAG	19	120398553	120398571	rev
GLDNrev	GCCAGGGCAGCCTTTATATG	20	120398934	120398915	rev
DistUTR2for1	CCGCGCATCATTAGCAAAACT	21	120477287	120477307	for
DistUTR2rev2	GCGGCTGTTTAAGAACCGGT	20	120477290	120477271	rev
DistUTR2for2	CATCATTAGCAAAACTCACCAT	22	120477292	120477313	for
DistUTR2rev1	ATGCGCGGCTGTTTAAGAAC	20	120477294	120477275	rev
ProxUTRfor	CAAATATGTCTTGTCTAAGTGTCCA	25	120525069	120525093	for
CDSrev	TTGCAATGCTGCCAAAAAGGA	21	120525325	120525305	rev
CYP19A3_for	CCTCTGGAAAGCTGTTCGACCTTTC	25	120541804	120541828	for
CYP19A1_for	CCTCTGGAAAGCTGTTAGAACTTAT	25	120611584	120611608	for
CYP19A2_for	CCTCTGGAAAGCCGTTAGAACTTAC	25	120674035	120674059	for
CYP19rev	GTAGCCCAAGTCATTGCGG	19	120677180	120677162	rev

## Data Availability

The datasets used and/or analyzed during the current study are available from the corresponding authors on reasonable request.
